# The Photodynamic Antibacterial Effects of Silicon Phthalocyanine (Pc) 4

**DOI:** 10.3390/ijms16047851

**Published:** 2015-04-08

**Authors:** Matthew L. Dimaano, Chantal Rozario, Michelle M. Nerandzic, Curtis J. Donskey, Minh Lam, Elma D. Baron

**Affiliations:** 1 Department of Dermatology, Case Western Reserve University, Cleveland, OH 44106, USA; E-Mails: matthew.dimaano@case.edu (M.L.D.); rozario.4@buckeyemail.osu.edu (C.R.); minh.lam@case.edu (M.L.); 2 Research Service, Geriatric Research Education and Clinical Center, Cleveland Veterans Affairs Medical Center, Cleveland, OH 44106, USA; E-Mails: michellenerandzic@gmail.com (M.M.N.); curtis.donskey@va.gov (C.J.D.); 3 Department of Dermatology, Cleveland Veterans Affairs Medical Center, Cleveland, OH 44106, USA

**Keywords:** photodynamic therapy, silicon phthalocyanine 4, MRSA

## Abstract

The emergence of antibiotic-resistant strains in facultative anaerobic Gram-positive coccal bacteria, such as methicillin-resistant *Staphylococcus aureus* (MRSA), is a global health issue. Typically, MRSA strains are found associated with institutions like hospitals but recent data suggest that they are becoming more prevalent in community-acquired infections. It is thought that the incidence and prevalence of bacterial infections will continue to increase as (a) more frequent use of broad-spectrum antibiotics and immunosuppressive medications; (b) increased number of invasive medical procedures; and (c) higher incidence of neutropenia and HIV infections. Therefore, more optimal treatments, such as photodynamic therapy (PDT), are warranted. PDT requires the interaction of light, a photosensitizing agent, and molecular oxygen to induce cytotoxic effects. In this study, we investigated the efficacy and characterized the mechanism of cytotoxicity induced by photodynamic therapy sensitized by silicon phthalocyanine (Pc) 4 on (a) methicillin-sensitive *Staphylococcus aureus* (MSSA) (ATCC 25923); (b) community acquired methicillin-resistant *Staphylococcus aureus* (CA-MRSA) (ATCC 43300); and (c) hospital acquired methicillin-resistant *Staphylococcus aureus* (HA-MRSA) (PFGE type 300). Our data include confocal image analysis, which confirmed that Pc 4 is taken up by all *S. aureus* strains, and viable cell recovery assay, which showed that concentrations as low as 1.0 μM Pc 4 incubated for 3 h at 37 °C followed by light at 2.0 J/cm^2^ can reduce cell survival by 2–5 logs. These results are encouraging, but before PDT can be utilized as an alternative treatment for eradicating resistant strains, we must first characterize the mechanism of cell death that Pc 4-based PDT employs in eliminating these pathogens.

## 1. Introduction

The rapid replication of bacteria in combination with the occurrence of mutations that improve bacterial survival in the presence of antibiotics results in highly resistant bacterial strains. Methicillin-resistant *Staphylococcus aureus* (MRSA) is the most common pathogen of hospital-associated infections in the United States [1]. In 2011, the Centers for Disease Control and Prevention (CDC) reported that MRSA caused 80,000 infections and more than 11,000 deaths in the United States alone [2]. Additionally, clinicians have observed a gradual increase in MRSA infections in health care facilities [3]. Specifically, the rise of community associated methicillin resistant *Staphylcocus aureus* (CA-MRSA) is especially alarming as these infections occur in healthy individuals [4] and have been reported in large number [5].

Despite recent reports that have demonstrated an increase in the minimum inhibitory concentrations (MICs) for vancomycin, implying that the normal doses may no longer reach its fullest potential [6,7], vancomycin remains the current chosen treatment for MRSA [8]. Consequently, MRSA has evolved strains with a lower susceptibility or complete resistant to vancomycin [9,10,11]. In addition, resistance also occurred with another commonly used antibiotic, Linezoid [12,13]. Thus, alternative approaches have been actively investigated. For instance, the antimicrobial effects of photodynamic therapy (PDT) have been shown to be a promising modality to combat the recalcitrant bacterial infections [14].

PDT entails the interaction of light, a photosensitizing drug, and molecular oxygen to induce a biological response [15]. Originally known for its anticancer modalities, PDT is now expanded to treat non-malignant diseases that include age-related macular degeneration of the retina, which is characterized by uncontrollable growth of vascular tissue, and dermatopathological conditions, such as psoriasis [16]. Diseases that are associated with cellular hyperproliferation are excellent candidates for PDT as evidenced by cancer, and more recently in noncancerous, as well as dermatological conditions applications. Photosensitizers are known to preferentially accumulate in cells that are actively dividing and pathogenic agents grow at much faster rate than the mammalian cells [17]. Although photosensitizers are also known to accumulate in normal healthy cells, the photodynamic effect can be targeted specifically to unwanted cells by anatomically confining the delivery of the light source. Therefore, the overall toxicity or adverse effects to the host can be reduced [18].

## 2. Results

### 2.1. Pc 4 Uptake by S. Aureus

**Figure 1:**
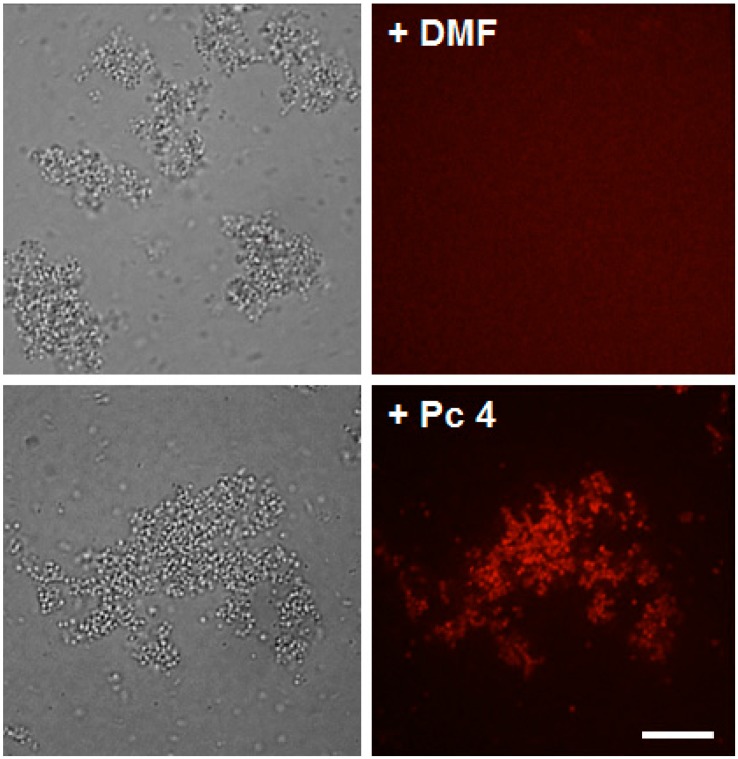
Phthalocyanine (Pc) 4 uptake in *S. aureus*. Bacteria were loaded with either 1.0 μM Pc 4 (**top** panels) or (vehicle control) *N*',*N*'-dimethylformamide (DMF) (**bottom** panels) overnight. Pc 4 (*pseudo red*) fluorescence appears to be taken up efficiently in HA-MRSA and equally comparing to the other two strains, MSSA and CA-MRSA (data not shown), within 3 h of incubation. Scale bars, 200 μm.

To substantiate the PDT effect in *S. aureus*, we examined the uptake of phthalocyanine (Pc) 4 by confocal microscopy. As shown in Figure 1, the vast majority of Pc 4 fluorescence could be seen on the cell wall as well as in the cytoplasm of the HA-MRSA after 3 h of incubation. Similar Pc 4 uptake was also found in both MSSA and CA-MRSA (data not shown).

### 2.2. Toxicity Studies

**Figure 2:**
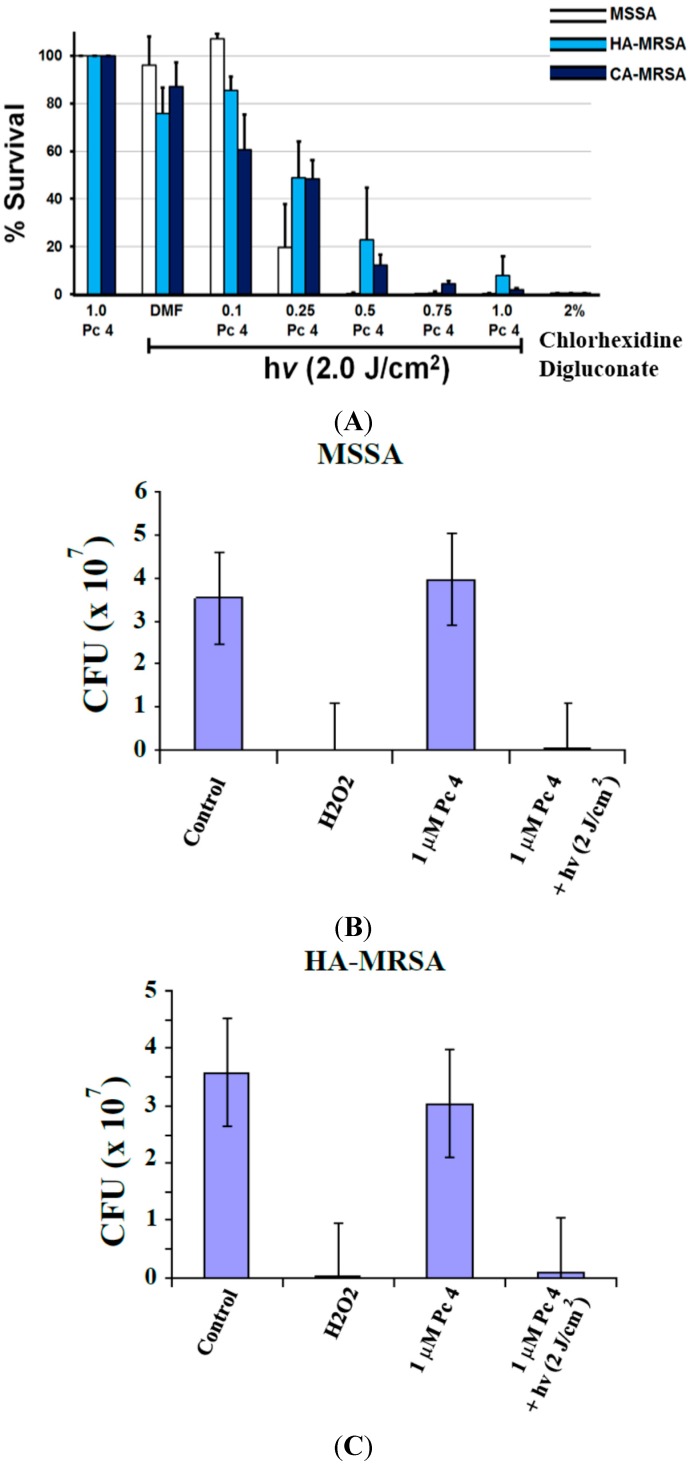

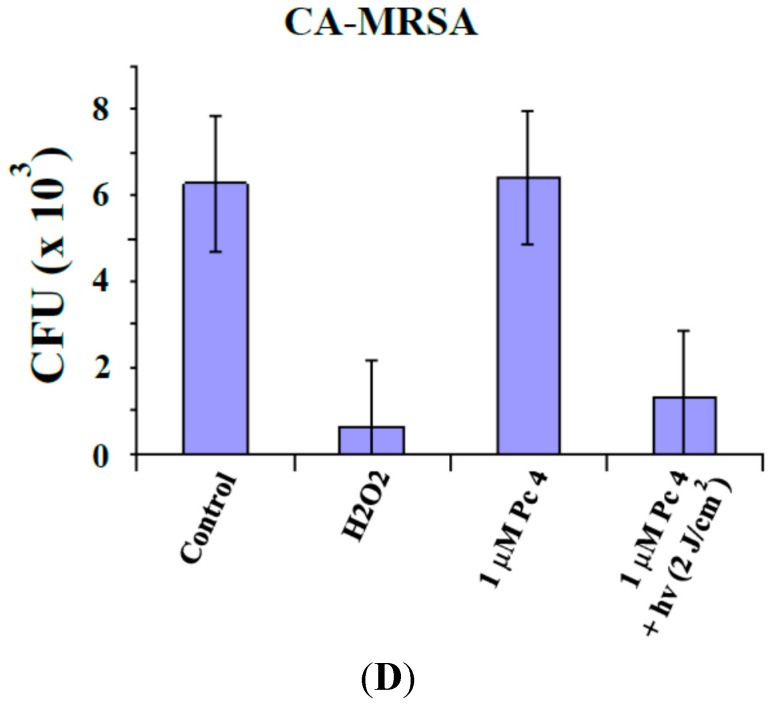
Pc 4-PDT kills *S. aureus* and inhibits colony formation. Bacteria cultures at equal concentrations were treated with various Pc 4 concentrations (100, 250, 500, 750, and 1000 nM) and incubated for 3 h and then followed by photoirradiation (see Experimental Section). The percentage of surviving cells from the PDT-treated cultures (MSSA, HA-MRSA and CA-MRSA) was normalized against cultures exposed to Pc 4 or light alone (**A**); The three strains are significantly affected by one single dose of Pc 4-PDT (1.0 µM and 2.0 J/cm^2^) as indicated by counting colony formation assays (**B**–**D**). Values represent the mean from at least three independent experiments. Error bars indicate S.E. For all Pc 4-PDT-treated cultures *vs.* untreated cells, either light alone or Pc 4 alone, *p* < 0.005.

**Figure 3:**
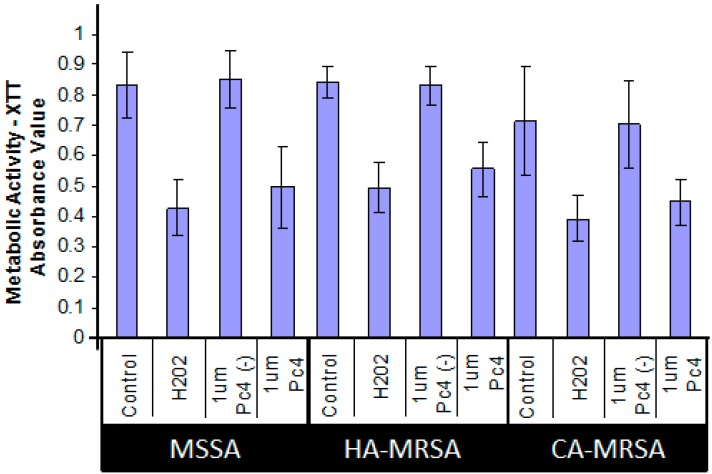
Cytotoxic effects, as measured by XTT, of Pc 4-PDT in MSSA, HA-MRSA and CA-MRSA. Equal quantities of three *S. aureus* culture strains were treated overnight with 1.0 μM Pc 4 and then were irradiated with 2.0 J/cm^2^ of 670–675 nm light. One hour following irradiation of Pc 4-loaded *S. aureus*, cultures were assayed for XTT reduction. H_2_O_2_ was used for a positive control. Values represent mean ± S.E. from at least three independent experiments. For all Pc 4-PDT-treated cultures, *p* < 0.005 *vs.* light alone control.

Colony Formation Unit (CFU) reduction—having established that Pc 4 can be taken up by *S. aureus*, we next determined a dose of Pc 4-PDT that would efficiently kill cells. All three strains of *S. aureus* were loaded with 0.1 to 1.0 µM Pc 4 for at least 3 h, then photoirradiated and plated. With a constant fluence (2.0 J/cm^2^), CFU data showed a dose response effect to increasing concentrations of Pc 4. Interestingly, at 1.0 µM Pc 4 treatment almost completely eliminated all strains ability to form colonies (Figure 2 and Figure 3).

XTT (2,3-*bis*[2-methoxy-4-nitro-5-sulfophenyl]-2*H*-tetrazolium-5-carboxyanilide) assay—using its cytotoxic dose, we then assessed the ability of Pc 4-PDT affecting the metabolic activities of the three strains of *S. aureus*. Following Pc 4-PDT, a decrease in absorbance, as demonstrated by a loss of water-soluble orange formazan derivative in the XTT assays, was indicative of metabolic impairment. As shown in Figure 3, metabolic activity was attenuated by ~50% within 4 h, compared to controls following photoirradiation of Pc 4-loaded MSSA, HA-MRSA and CA-MRSA.

### 2.3. ROS Generation Immediately Following Pc 4-PDT

**Figure 4:**
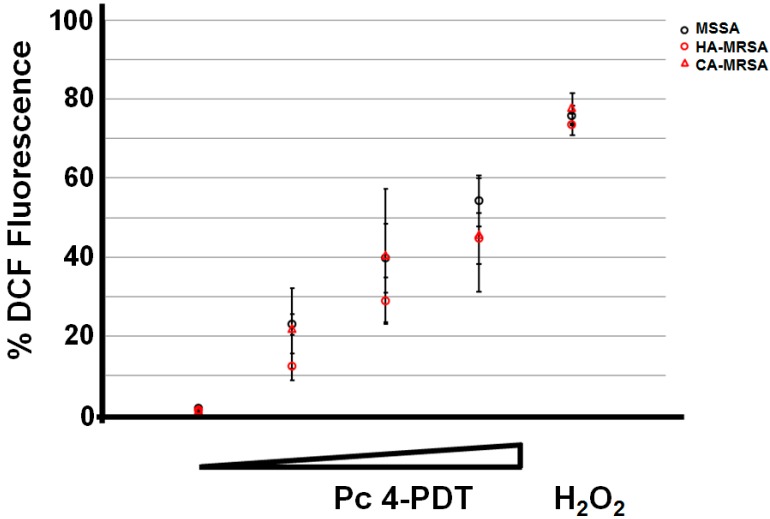
To monitor ROS generation by confocal microscopy and flow cytometry, Pc 4-treated *S. aureus* strains were loaded with 2',7'-dichlorofluorescin, as described in “Materials and Methods”. The generation of ROS based upon DCF fluorescence can be quantified by flow cytometry. For each sample, 100,000 events were collected on an Accuri C6 flow cytometer at 0, 6, 12, and 17 min during the light treatment, which yielded the following fluences 0, 7, 1.4, and 2.0 J/cm^2^, respectively. For a positive control, H_2_O_2_ was used. Results are expressed as mean ± S.E.M. from three independent experiments.

We demonstrated the formation of ROS with MSSA, HA-MRSA and CA-MRSA following Pc 4-PDT. To monitor the intracellular ROS production, we performed a dose-response study (Pc 4 at a concentration of 1.0 µM and fluence ranging from 0, 7, 1.4, and 2.0 J/cm^2^) by flow cytometry. Our data consistently demonstrated that a higher Pc 4-PDT dose correlated with a greater DCF fluorescence or ROS level in all MSSA, HA-MRSA and CA-MRSA (Figure 4). As a positive control, we used H_2_O_2_ (53.27 mM) to show the enhanced level of ROS in all strains of *S. aureus*. Red light alone had no effect on DCF fluorescence (data not shown).

## 3. Discussions

PDT has been known for its versatility in treating various medical conditions, ranging from age-related macular degeneration to psoriasis to malignant cancers [19]. In recent years, PDT has also been attracting considerable attention for its antimicrobial properties [20]. PDT is based on the utilization of photosensitizers, which can accumulate in specifically selected tissues or cells. In the present of molecular oxygen, the photosensitizers can be activated by light with an appropriate wavelength that leads to the formation of singlet oxygen and free radicals for the cytotoxic effects [15].

It has been reported that PDT using different photosensitizers, such as hematoporphyrin, photofrin, 5-aminolaevulinic acid (ALA) and phthalocyanine, can lead to cytotoxicity in *S. aureus* [21]. The silicon phthalocyanine (Pc) 4, which is currently undergoing two separate clinical trials, for cutaneous T cell lymphoma and psoriasis, has also been shown to have antimicrobial effects in yeast-like fungi pathogens, such as *C. albicans* and *T. rubrum* *in vitro* [22,23]. Although a currently FDA-approved photosensitizer, ALA, has demonstrated an antifungal effect in *T. rubrum* *in vitro*, its limitation is the need for bioconversion to PpIX [24] prior to irradiation. Unlike ALA, Pc 4 does not require the bioconversion step. In other words, upon treatment, its photocytotoxicity effect occurs as soon as Pc 4 is exposed to 670–675-nm light.

In the current study, we demonstrated that Pc 4 is found taken up by all strains of *S. aureus* by confocal microscopy. In addition, ROS formation and metabolic impairment is detected immediately following Pc 4-PDT, which subsequently leads to cell death. In summary, data from this study indicate that Pc 4-PDT has potential antimicrobial effects in multiple strains of MRSA by disrupting overall metabolic activity and resulting in cytotoxicity. Based upon these findings, the development of Pc 4-PDT as a potential clinical antimicrobial therapy, or as an adjunctive therapy with a currently approved antibiotic agent, warrants further exploration.

## 4. Experimental Section

### 4.1. Bacteria

*Staphylcoccus aureus* strains MSSA (ATCC 25923, Manassas, VA, USA), CA-MRSA (ATCC 43300) and HA-MRSA (PFGE type 300) were used in this study. Bacteria was streaked onto BBL™ Trypticase™ Soy Agar with 5% Sheep Blood (TSA II) (BD Biosciences, Franklin Lakes, NJ, USA) and incubated overnight at 37 °C. Bacterial growth was collected with a sterile loop and suspended in 1× PBS. The OD_600_ for the solution was determined using a spectrophotometer (Spectronic Genesys 5; Analytical Instruments, Golden Valley, MN, USA). The OD_600_ for all experiments was 0.4. Calculation of the OD was done according to the formula (Available online: http://2011.igem.org/wiki/images/0/0c/OD600_100.pdf):

(1) $$\text{X}=\text{Current volume}\times \frac{\left({\text{Current OD}}_{600}-1\right)}{\text{0.4}}$$

where X is the volume of 1× PBS to be added to bacterial suspension.

### 4.2. Pc 4-Photodynamic Treatment Conditions

Pc 4 in its powder form was dissolved in a vehicle of *N*',*N*'-dimethylformamide (DMF, ThermoFisher, Waltham, MA, USA) to 0.5 mM and stored at 4 °C in the dark. *Bacteria* cultures were incubated with Pc 4 concentrations ranging from 0.1 to 1.0 μM in 1× PBS containing 10% fetal bovine serum (FBS) for at least 3 h at 37 °C in the dark and subsequently irradiated with red light using a light-emitting diode array (EFOS, Mississauga, ON, Canada) at a fluence ranging from 1.0 to 2.0 J/cm^2^ (1.0 mW/cm^2^, λ_max(Ex)_~670–675 nm) at room temperature.

### 4.3. Confocal Microscopy

To visualize the uptake of Pc 4, bacteria in 1× PBS supplemented with 10% FBS were loaded with 1 μM Pc 4 and incubated for at least 3 h. All images were acquired using an UltraView VoX spinning-disc confocal system (PerkinElmer, Waltham, MA, USA) mounted on a Leica DMI6000B microscope (Leica Microsystems, Inc., Bannockburn, IL, USA) equipped with an HCX PL APO _100x/1.4 oil immersion objective. Confocal images of Pc 4 fluorescence were collected using solid-state diode 640-nm and with a 650-nm emission filter.

### 4.4. Viable Cell Recovery Assay

ChromAgar plates were made according to manufacturer instructions (Chromagar, Paris, France) with the addition of 8 µg/mL of cefoxitin to select for MRSA and exclude MSSA. *S. aurueus* strains were treated with various concentrations of Pc 4 (100 nM, 250 nM, 500 nM, 750 nM and 1 µM) and incubated for 3 h. Chlorohexidine was used as a positive control as it is often used in health care facilities to eradicate MRSA. Chlorhexidine digluconate was added to samples so that the final concentration was 2% by volume and incubated concurrently with Pc 4 for 2 h. After light treatment, samples were pipetted into 96 well plate and serially diluted 10-fold in PBS to 10^−6^ of the original concentration. Dilutions were then drop-plated onto ChromAgar allowed to rest for 30 min so the liquid could settle into the agar, and incubated for 24 h at 37 °C before counting by eyes.

### 4.5. Metabolic XTT Assay

Metabolic activity was assayed using the colorless sodium salt of XTT (2,3-*bis*[2-Methoxy-4-nitro-5-sulfophenyl]-2*H*-tetrazolium-5-carboxyanilide inner salt) (Sigma-Aldrich, St. Louis, MO, USA), which is converted by mitochondrial dehydrogenases of viable cells. Ninety-six-well plates were pipetted with 100 μL of *S. aureus* suspension in PBS at OD_600_ of 0.5, 12.5 μg/mL XTT and 1 µM menadione were added to PBS in a separate conical tube. 100 µL of the XTT/Menadione solution was added to wells with *S. aureus* suspended in PBS and incubated in 37 °C incubator for 3 h. After incubation, the plate was spun down 4000 RPM for 5 min. One hundred microliters of the supernatant XTT solution was collected and transferred to another 96 well plate. Absorbance values were measured with a Versamax microplate reader (Molecular Devices, Sunnyvale, CA, USA) set to 492 nm wavelength.

### 4.6. Flow Cytometry

To quantitatively measure reactive oxygen species (ROS) formation [25], CA-MRSA, HA-MRSA, and MSSA were treated with 1 µm Pc 4 for 3 h in 1× PBS with 10% FBS. Then the bacteria was resuspended in flow buffer which contained 5% FBS and 2 mM EDTA in 1x PBS and stained with 50 µM DCFDA (2',7'-dichlorodihydrofluorescein diacetate) (Invitrogen, Carlsbad, CA, USA) and incubated on ice for 90 min. Events were recorded using an Accuri C6 flow cytometer (BD, Franklin Lakes, NJ, USA) at 0, 6, 12, and 17 min during the light treatment which correspond to 0, 7, 1.4, and 2.0 J/cm^2^ fluences, respectively.
